# Significance of busulfan administration route including therapeutic drug monitoring in the conditioning regimen of pediatric patients prior to hematopoietic stem cell transplantation

**DOI:** 10.1007/s00432-025-06179-w

**Published:** 2025-04-04

**Authors:** Stephanie Hämmerle, Jana Ernst, Regula Steiner, Tayfun Güngör, Till Milde, Bernd Gruhn

**Affiliations:** 1 Department of Pediatrics, Jena University Hospital, Am Klinikum 1, 07747 Jena, Germany; 2 Comprehensive Cancer Center Central Germany (CCCG), Jena, Germany; 3 Institute of Clinical Chemistry, University and University Hospital of Zurich, Zurich, Switzerland; 4 Division of Stem Cell Transplantation and Children’S Research Center, University Children’S Hospital Zurich, University of Zurich, Zurich, Switzerland; 5 Hopp Children’S Cancer Center Heidelberg (Kitz), Heidelberg, Germany; 6 Clinical Cooperation Unit Pediatric Oncology, German Cancer Research Center Heidelberg (DKFZ), Heidelberg, Germany

**Keywords:** Busulfan, Therapeutic Drug Monitoring, Hematopoietic stem cell transplantation, Pediatric, Overall survival, Transplantation-related mortality, Hepatic sinusoidal obstruction syndrome

## Abstract

**Purpose:**

Busulfan is an important myeloablative agent in various conditioning regimens prior to hematopoietic stem cell transplantation (HSCT) in pediatric patients. This retrospective study compares three different routes of busulfan administration and their impact on transplantation-related mortality (TRM) and overall survival (OS).

**Methods:**

The study included 250 pediatric patients with malignant and non-malignant diseases who underwent HSCT at the Department of Pediatrics, Jena University Hospital, Jena, Germany. One hundred forty-eight patients received busulfan orally without therapeutic drug monitoring (TDM) (group 1), 62 patients received busulfan intravenously (i.v.) without TDM (group 2) and 40 patients received busulfan i.v. with additional TDM (group 3).

**Results:**

The TRM rate at 5 years after transplantation for all patients was 40.5% for group 1, 25.2% for group 2, and 8.4% for group 3 (*p* < 0.001). The TRM rate at 5 years after transplantation for patients with malignant diseases only was 40.3% for group 1 compared to 28.4% for group 2 and 15.3% for group 3 (*p* = 0.051). For patients with non-malignant diseases, group 1 showed a TRM rate of 43.8% compared to 15.4% in group 2 and 4.6% in group 3 (*p* = 0.009). In addition, the 5-year OS rate for all patients was 39.9% for group 1, 61.2% for group 2, and 83.9% for group 3 (*p* < 0.001). Regarding the OS of the groups for patients with only malignant or only non-malignant diseases, we obtained similar results with *p*-values of *p* = 0.017 and *p* = 0.007, respectively. The cumulative incidence of hepatic sinusoidal obstruction syndrome (SOS) for patients with malignant diseases and a cumulative AUC > 85.0 mg/L x h was 55.6%, while patients with malignant diseases and a cumulative AUC < 85.0 mg/L x h showed a cumulative incidence of 11.1% (*p* = 0.038).

**Conclusion:**

In this study, we demonstrate that patients with i.v. administration of busulfan with TDM had a significantly lower rate of TRM and a significantly improved OS compared to patients who received i.v. administration of busulfan without TDM, who, in turn, had a better outcome than patients with oral busulfan administration. Additionally, these data emphasize the clinical relevance of AUC measurements in patients with malignant diseases to prevent hepatic SOS.

## Introduction

Busulfan is one of the most frequently administered myelotoxic agents for conditioning regimen prior to hematopoietic stem cell transplantation (HSCT). Santos et al. introduced it in the early eighties to replace total body irradiation (TBI) (Bartelink et al. 2008; Santos et al. 1983; Socié et al. 2001). Busulfan is an alkylating agent that interacts with DNA and RNA single strands. By cross-linking bases, busulfan damages DNA replication and thus inhibits proliferating cells. Despite its efficacy, busulfan has multiple adverse effects, e.g. the induction of hepatic endothelial damage leading to a sinusoidal-obstruction syndrome (SOS) of the liver (Grochow et al. 1990), particularly prevalent among young children. Due to this important toxicity for endothelial cells of the liver, busulfan has a small therapeutic range (Schiltmeyer et al. 2003).

When busulfan is administered orally, it shows high intra- and interindividual variability based on age, liver function and pharmacokinetics (PK) (Malär et al. 2011). PK is important in pediatric patients, as it diverges from the PK of older patients, requiring different dosage schedules (Oechtering et al. 2005; Zao et al. 2015).

Overdosing as well as underdosing can have an adverse effect on prognosis (Malär et al. 2011). While overdosing results in higher toxicity and a higher incidence of transplantation-related mortality (TRM), underdosing resulting in loss of efficacy can cause a higher rate of relapse, mixed chimerism and graft failure (Bolinger et al. 2000). Benadiba et al. (2018) emphasizes the variability in busulfan PK as a risk factor for toxicity and graft failure.

To overcome the variability in busulfan levels upon oral administration, intravenous (i.v.) administration of busulfan was introduced in 1999 (McCune et al. 2009). The dose of busulfan administered was based on the patient’s bodyweight (Nguyen et al. 2004; Scott et al. 2012). Although in theory, variability of busulfan levels should have been reduced, variability persisted, leading to the implementation of therapeutic drug monitoring (TDM) (McCune et al. 2000).

TDM is a method to determine busulfan plasma levels in blood samples taken after the first and second administration. With these plasma levels the area-under-the-curve (AUC) is calculated (Malär et al. 2011). According to the underlying disease, the target cumulative AUC varies (Bartelink et al. 2016). Some patients who could not reach the target cumulative AUC with the initially planned doses due to age, clearance and other factors needed individual dose adjustments to optimize outcomes (Chattergoon et al. 1997).

Until now, there are no clear recommendations for the target cumulative AUC, depending on the underlying disease and other factors such as age and the conditioning regimen used (Shao et al. 2022). Feng et al. (2020) suggest an AUC greater than 900 µM x min (cumulative AUC of 59.1 mg/L x h) to avoid graft failure and a cut-off AUC less than 1350 µM x min (cumulative AUC of 88.7 mg/L x h) to prevent hepatic SOS. However, Bartelink et al. (2016) suggest an AUC range between 78 mg/L x h and 101 mg/L x h for the highest event-free survival.

Although Kashyap et al. (2002) demonstrated that i.v. administration of busulfan alone is associated with better outcomes after HSCT, there are authors claiming that i.v. administration steered by TDM may further improve outcomes of patients compared to oral administration and i.v. administration without TDM. Shimoni et al. (2003) suggested that TDM could be particularly beneficial for patients with a high risk of relapse under the current regimen, such as patients with advanced malignancies.

This retrospective study aims to compare three routes of busulfan administration, evaluating outcome regarding transplantation-related mortality (TRM) and overall survival (OS) in pediatric patients conditioned with busulfan prior to HSCT.

### Patients and methods

#### Study design and patients

This retrospective, non-randomized, single-center study includes 250 pediatric patients with malignant and non-malignant diseases who underwent allogeneic or autologous HSCT at the Department of Pediatrics, Jena University Hospital, Jena, Germany. All patients received a busulfan based conditioning regimen. Busulfan was administered orally in 148 patients (group 1). Sixty-two patients received busulfan i.v. without TDM (group 2) and 40 patients received busulfan i.v. with additional TDM (group 3). Table 1 describes clinical characteristics of the patients.

**Table 1:** Characteristics of patients (n = 250)

Characteristics	Total no. (%)
Median age of the patients (years)	8.5
Sex Male Female	166 (66.4) 84 (33.6)
Type of HSCT Allogeneic HSCT Autologous HSCT	190 (76.0) 60 (24.0)
Disease AML/ MDS/ CMML ALL CML JMML NHL RBD NBL EWS G MYF GCT	114 (45.6) 40 (16.0) 11 (4.4) 7 (2.8) 6 (2.4) 4 (1.6) 9 (3.6) 6 (2.4) 51 (20.4) 1 (0.4) 1 (0.4)
Malignancy Malignant disease Non-malignant disease	199 (79.6) 51 (20.4)
Stage of disease Genetic disease Remission Relapse Primary disease Chronic phase (CML)	51 (20.4) 124 (49.6) 23 (9.2) 41 (16.4) 11 (4.4)
Donor type HLA-mismatched unrelated HLA-matched unrelated HLA-mismatched related HLA-matched related autologous	16 (6.4) 91 (36.4) 12 (4.8) 71 (28.4) 60 (24.0)

*AML* acute myeloid leukemia, *MDS* myelodysplastic syndrome, *CMML* chronic myelomonocytic leukemia, *ALL* acute lymphoblastic leukemia, *CML* chronic myeloid leukemia, *JMML* juvenile myelomonocytic leukemia, *NHL* non-Hodgkin lymphoma, *RBD* rhabdomyosarcoma, *NBL* neuroblastoma, *EWS* ewing sarcoma, *G* genetic disease, *GCT* germ cell tumor, *HSCT* hematopoietic stem cell transplantation, *MYF* myelofibrosis, *HLA* human leukocyte antigen

### Busulfan administration

Busulfan was administered orally at a fix dose of 1 mg/kg four times daily in a 6-h interval. The patients were treated with busulfan for four days, resulting in a cumulative dosage of 16 mg/kg. Busulfan formulation was only available as a 2 mg pill requiring patients to ingest a significant number of pills.

Busulfan was administered i.v. as a 2-h infusion four times daily every six hours for a total of 96 h. Nguyen et al. (2004) proposed a dosing regimen adjusted to the patient’s body weight. Patients with a body weight less than 9 kg, from 9 kg to less than 16 kg and from 16 kg to less than 23 kg received a single dose of 1 mg/kg, 1.2 mg/kg and 1.1 mg/kg, respectively. Lower doses of 0.95 mg/kg and 0.8 mg/kg were administered to children with a body weight from 23 to 34 kg and greater than 34 kg, respectively.

For i.v. busulfan with TDM, doses were administered twice daily as a 4-h infusion in a twelve-hours interval. The dosage suggested by Nguyen et al. (2004), based on the patient’s body weight, was doubled for one infusion and was then adjusted to the targeted cumulative AUC. The period of busulfan treatment, and thereby the total number of doses, varied depending on the AUC after the first and second dose and the patients’ disease. Doses were increased to achieve the target cumulative AUC if necessary. We aimed at a target cumulative AUC of 85.0 mg/L x h for patients with malignant diseases and 70.0 mg/L x h for patients with non-malignant diseases. The conditioning regimen included other cytotoxic agents like cyclophosphamide, fludarabine or melphalan.

### Busulfan therapeutic drug monitoring

The first administration of busulfan started at midnight. Blood samples were collected prior to the infusion and at 0 min, 30 min, 60 min, 120 min, 240 min and 360 min following the first and second infusion from a central catheter. For this, another lumen from the one used for busulfan administration was used. Lithium heparin tubes were used for blood collection. Then the blood collection tubes were centrifuged, and the obtained plasma was frozen.

The plasma was stored on dry ice and transported overnight to the Department of Clinical Chemistry, University Hospital Zurich, Zurich, Switzerland, where the measurements were performed.

Plasma was analyzed by high performance liquid chromatography-mass spectrometry to determine the busulfan concentration. This procedure was conducted for every blood sample. The AUC after the first and the second infusion was calculated by using these concentrations over time. Calculations were made using the program Win-Nonlin (version 5.2; Pharsight, Mountain View, CA, USA). The cumulative AUC was calculated according to the following formula:

$$AUCcum\,\, = \,AUC1\, + \,\left( {x \cdot \, * \cdot AUC2\,} \right)$$

where x represents the number of planned and administered additional doses.

Based on a malignant or non-malignant disease the target AUC varied. Doses were adjusted to achieve the target cumulative AUC. If necessary, typically more doses were administered; however, if the cumulative AUC could not be achieved within time by increasing the number of doses, the dose itself was also increased. Dose increments up to 30–40% were occasionally necessary.

### Endpoints

TRM was defined as the time from HSCT to death caused by any other causes than their underlying disease or relapse (e.g. infections, toxicity because of conditioning regimen, SOS). Patients who died of their underlying disease and patients who survived were censored at last follow up. OS was defined as the time from HSCT to death by any cause.

### Statistical analysis

The competing risk model was used to estimate the cumulative incidence of TRM. The Fine and Gray model was applied to assess statistical significance. We used the Kaplan–Meier method to estimate the OS and compared the calculations with the log-rank test. Calculations with p-values less than 0.05 were considered statistically significant. Multivariate analysis was used to identify any association between the method of busulfan administration and TRM, as well as OS. For this, a Cox-proportional hazard model was used. The results were demonstrated with corresponding p values (*p*), hazard ratio (HR) and 95%-confidence interval (CI). All statistical calculations were made using IBM SPSS Statistics Premium 29 for Windows and R-4.3.2.

## Results

### TRM and OS analysis

**Fig. 1:**
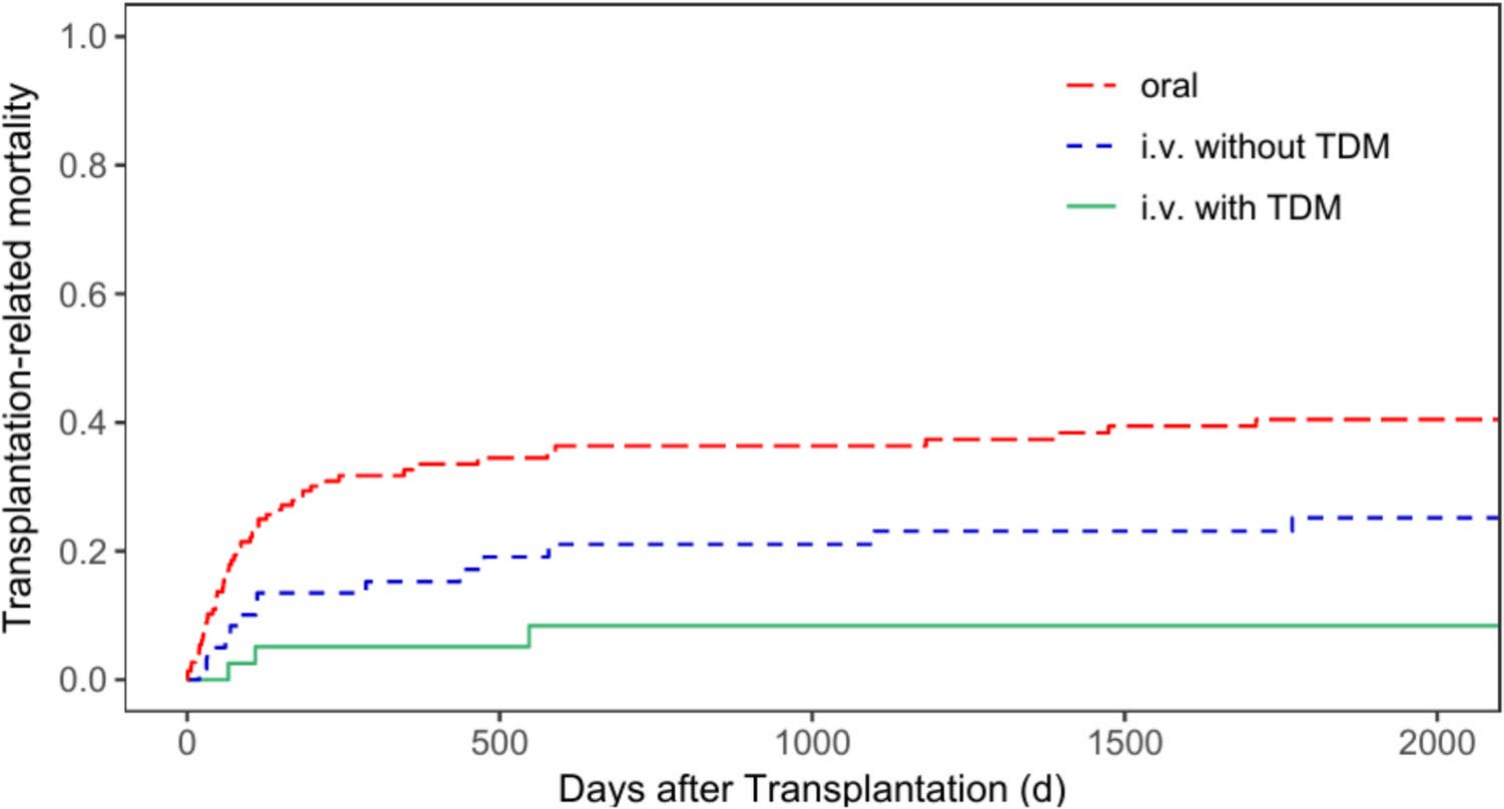
Transplantation-related mortality for all patients according to patients’ busulfan administration (p < 0.001)

We observed a statistically significant association between the route of busulfan administration and TRM. The cumulative incidence of TRM for all patients at five years after transplantation was 40.5% (95% CI = 31.8—48.9%) for group 1, 25.2% (95% CI = 14.5—37.3%) for group 2, and 8.4% (95% CI = 2.1—20.6%) for group 3 (*p* < 0.001, Fig. 1).

**Fig. 2:**
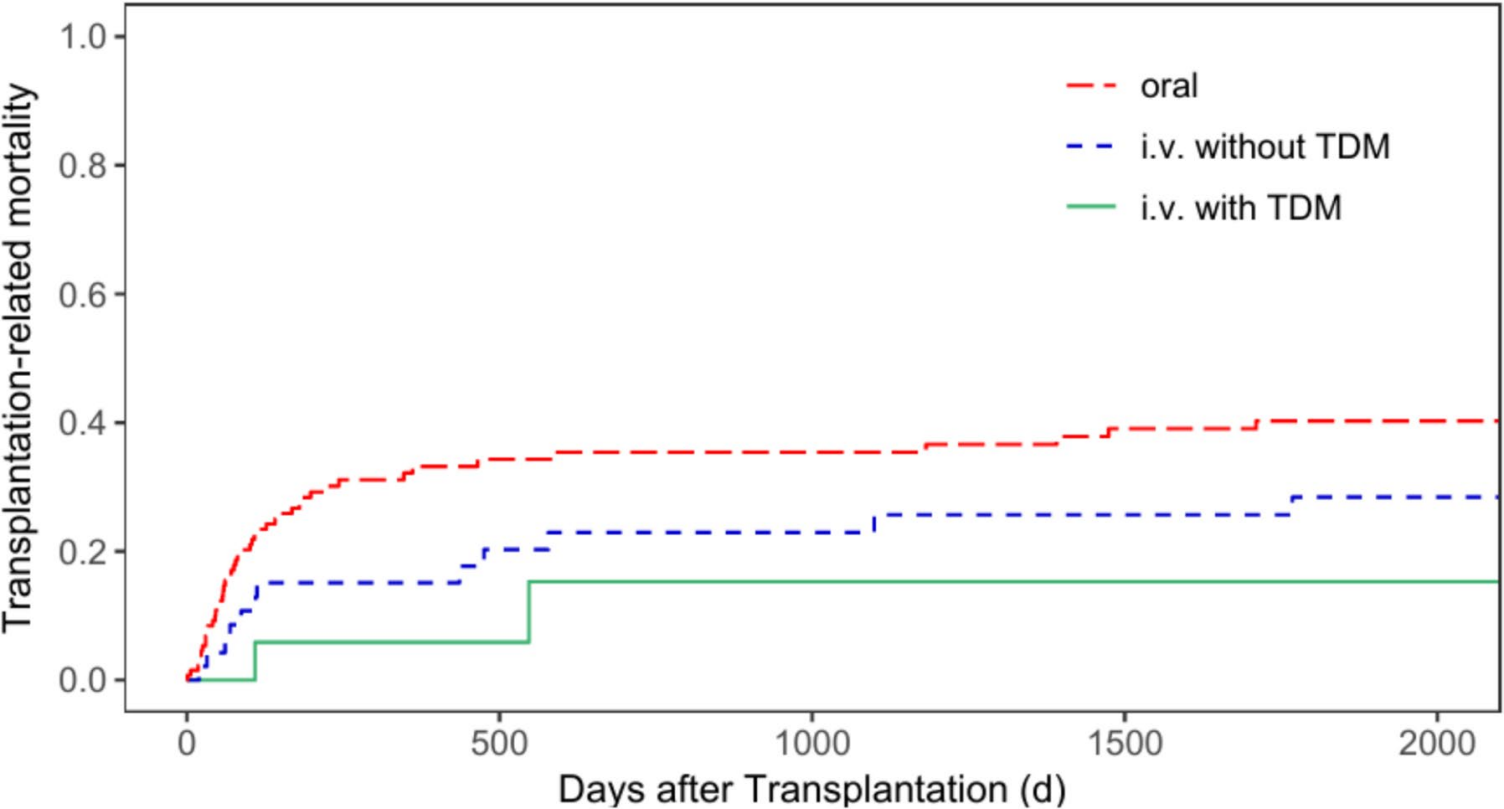
Transplantation-related mortality for patients with malignant diseases according to patients’ busulfan administration (p = 0.051)

**Fig. 3:**
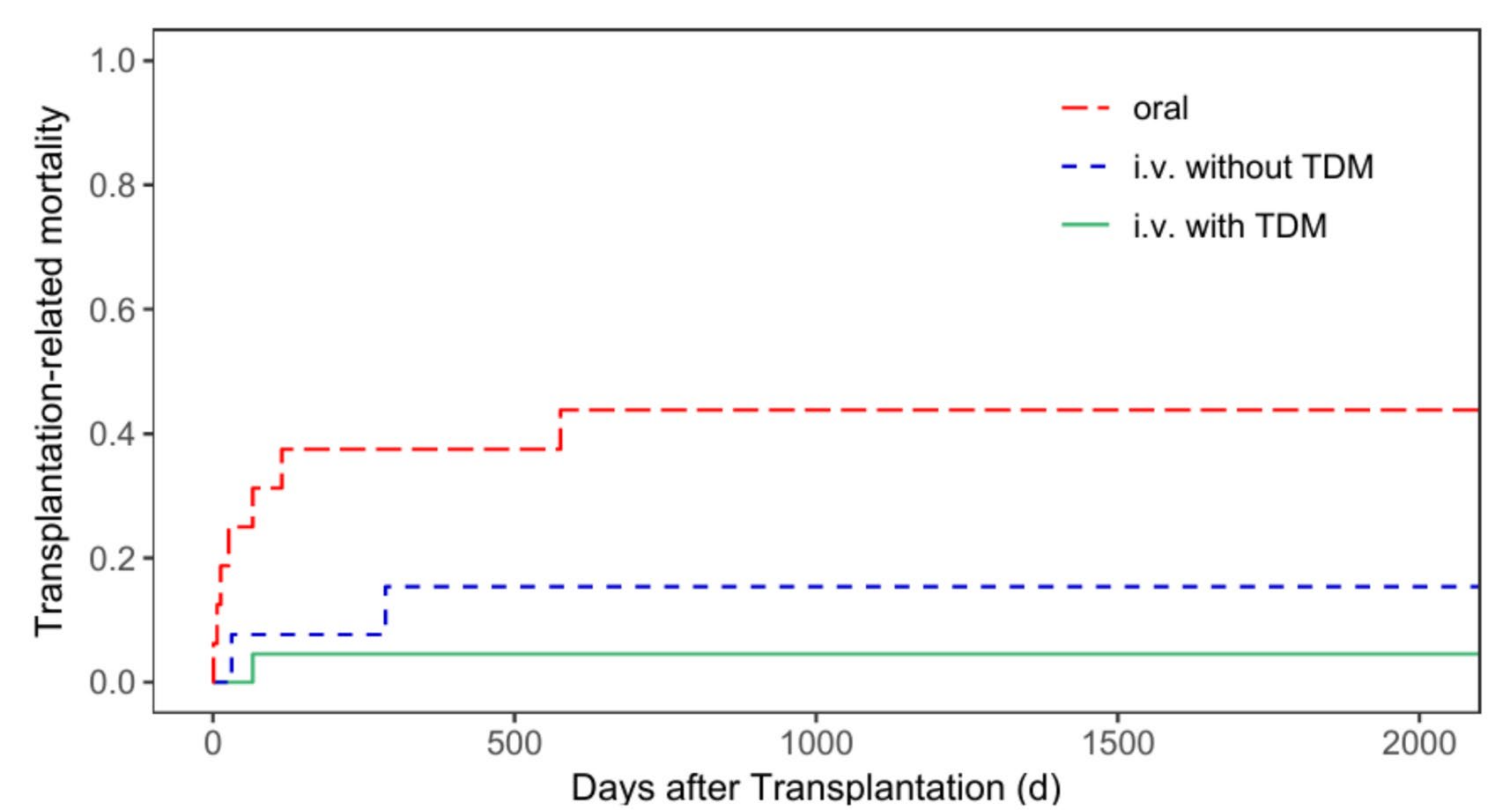
Transplantation-related mortality for patients with non-malignant diseases according to patients’ busulfan administration (p = 0.009)

The cumulative incidence of TRM for patients with malignant diseases only was 40.3% (95% CI = 31.0—49.4%) for oral administration, 28.4% (95% CI = 15.5—42.8%) for i.v. administration without TDM, and 15.3% (95% CI = 2.0—40.7%) for i.v. administration with TDM (*p* = 0.051, Fig. 2). The TRM rate for patients with non-malignant diseases only was 43.8% (95% CI = 18.9—66.4%) for group 1, 15.4% (95% CI = 2.2—39.8%) for group 2, and 4.6% (95% CI = 0.3—19.4%) for group 3 (*p* = 0.009, Fig. 3).

**Fig. 4:**
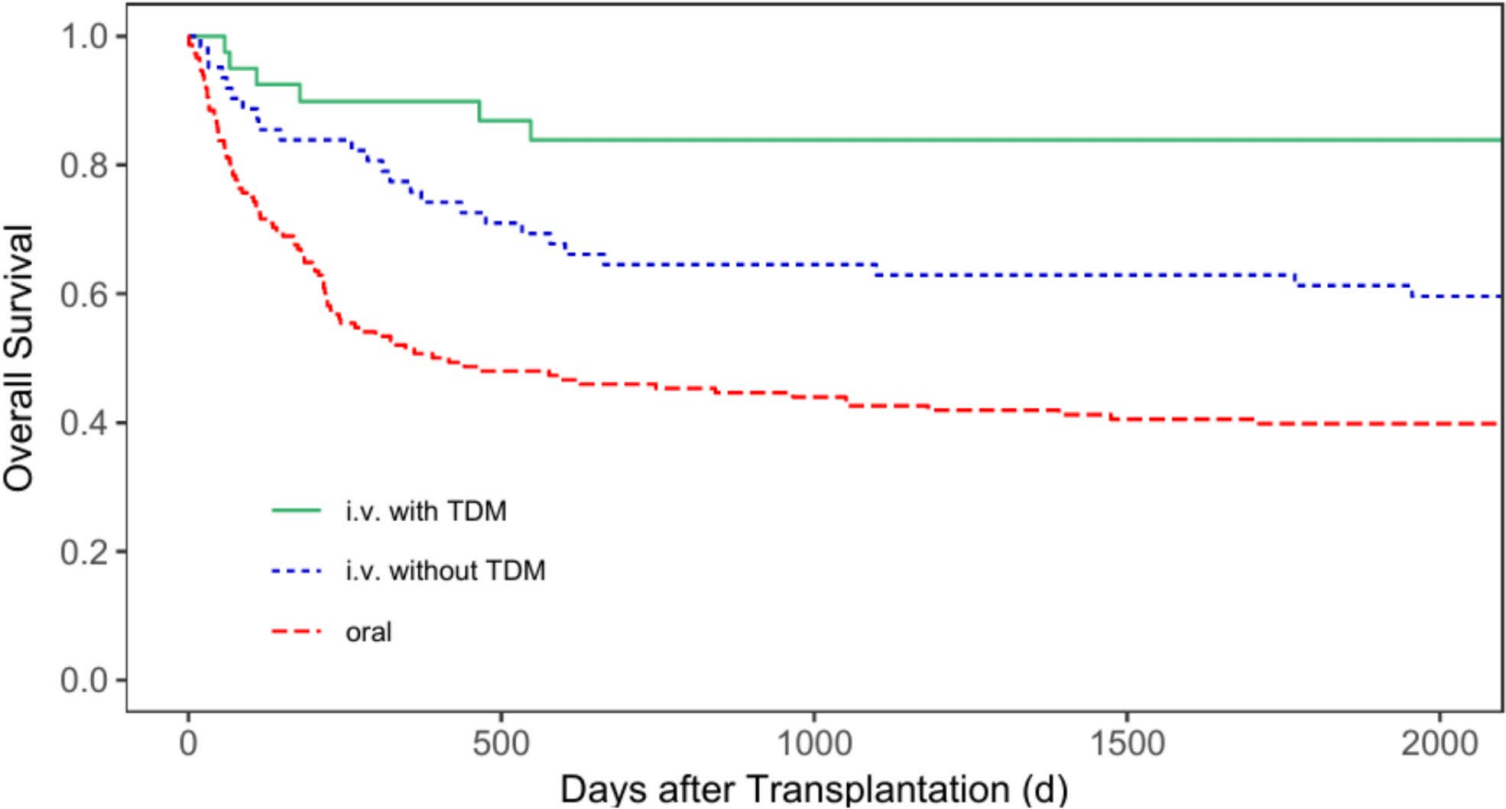
Overall survival for all patients according to the patients’ busulfan administration (p < 0.001)

**Fig. 5:**
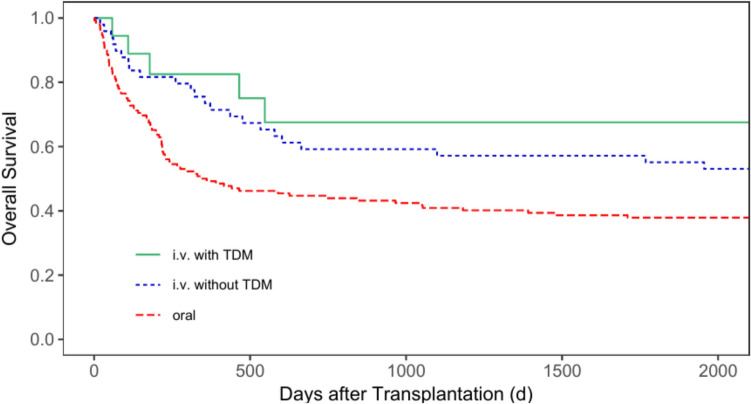
Overall survival for patients with malignant diseases according to patients’ busulfan administration (p = 0.017)

**Fig. 6:**
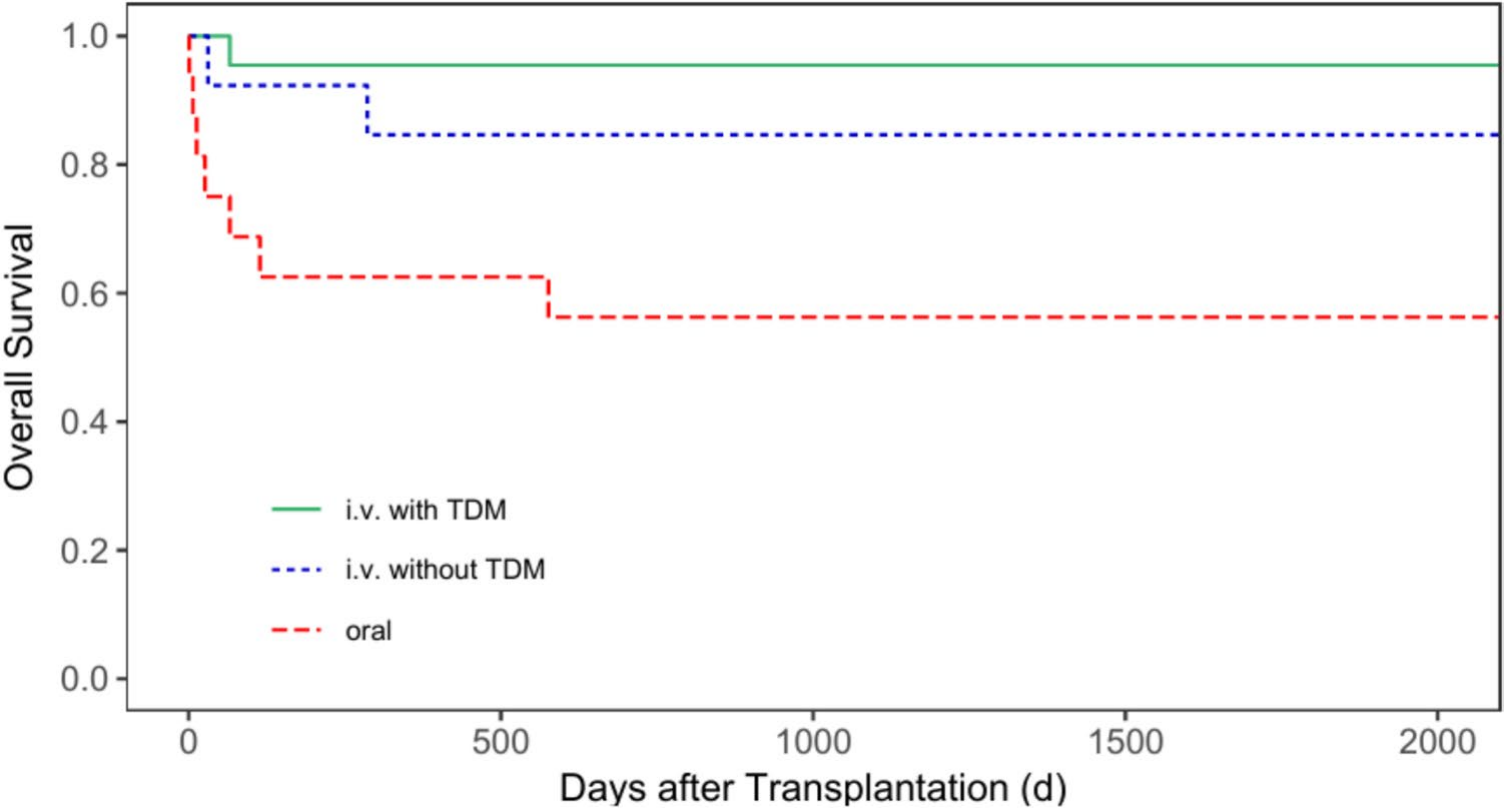
Overall survival for patients with non-malignant diseases according to patients’ busulfan administration (p = 0.007)

Regarding the five-year OS (5y-OS) after transplantation for all patients, the 5y-OS rate for patients receiving busulfan orally was 39.9% (95% CI = 32.7—48.6%), 61.2% (95% CI = 50.2—74.7%) for patients receiving busulfan i.v. without TDM, and 83.9% (95% CI = 72.8—96.7%) for patients with i.v. administration and TDM (*p* < 0.001, Fig. 4). The 5y-OS for patients with malignant diseases only was 37.9% (95% CI = 30.4—47.1%), 55.1% (95% CI = 42.8—70.9%) and 67.5% (95% CI = 47.4—96.2%) for group 1, 2 and 3, respectively (*p* = 0.017, Fig. 5). Regarding patients with non-malignant diseases only, group 3 also showed the best 5y-OS rate. Group 1 showed a 5y-OS rate of 56.3% (95% CI = 36.5—86.7%), compared to 84.6% (95% CI = 67.1—100.0%) for group 2, and 95.5% (95% CI = 87.1—100.0%) for group 3 (*p* = 0.007, Fig. 6).

### SOS analysis

**Fig. 7:**
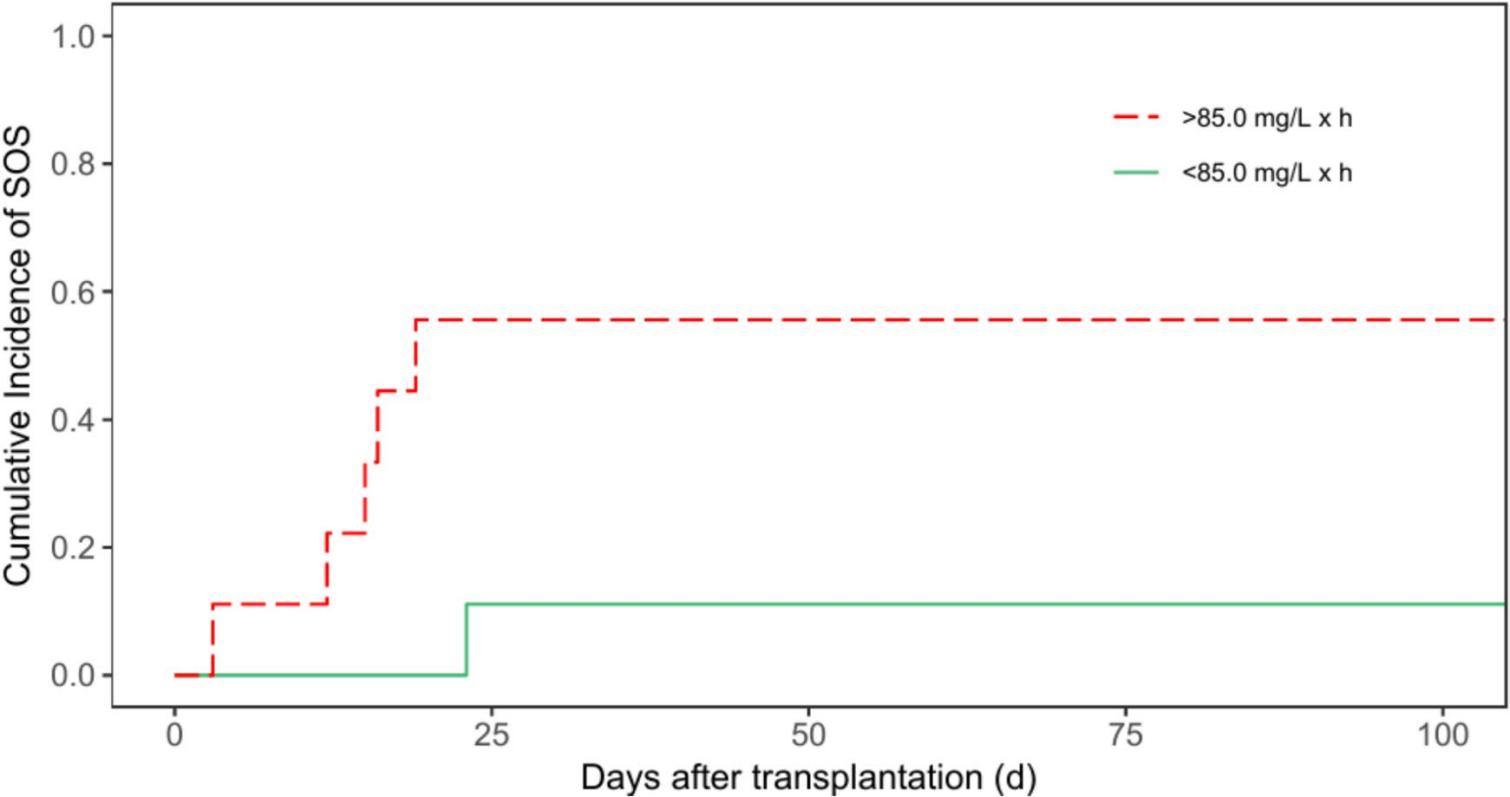
Cumulative Incidence of SOS for patients with malignant diseases according to patients’ cumulative area-under-the-curve (p = 0.038)

The criteria established by the European Society for Blood and Marrow Transplantation to diagnose SOS in pediatric patients were used (Corbacioglu et al. 2018). The cumulative incidence of hepatic SOS was analyzed for patients with malignant diseases only who received i.v. administration of busulfan with TDM. Patients with a cumulative AUC greater than 85.0 mg/L x h showed a cumulative incidence of hepatic SOS of 55.6% (95% CI = 17.5 – 82.0%) in comparison to 11.1% (95% CI = 0.47 – 40.6%) for patients with a cumulative AUC less than 85.0 mg/L x h (*p* = 0.038, Fig. 7).

### Multivariate analysis

A multivariate analysis was used to identify an association between the route of busulfan administration and other clinical factors influencing TRM and OS. To confirm that the route of busulfan administration is an independent prognostic factor, we integrated the following potential confounding variables: age, conditioning regimen, HLA compatibility, donor-recipient-gender match and disease risk (defined by the disease and its stage according to Arndt et al. (2014)). We confirmed that the route of busulfan administration as well as disease risk are independent risk factors for TRM and OS (*p* < 0.05). The results are presented in Table 2.

**Table 2:** Multivariate analysis. Transplantation-related mortality (TRM) and overall survival (OS)

	TRM		OS	
Variable	HR (95% CI)	*P*	HR (95% CI)	*P*
BU BU (group 1 vs. group 3) BU (group 2 vs. group 3) BU (group 1 vs. group 2)	4.224 (1.306–13.656) 2.636 (0.744–9.339) 1.602 (0.844–3.041)	**0.029** **0.016** 0**.**133 0.150	4.138 (1.789–9.570) 2.431 (0.981–6.027) 1.702 (1.050–2.758)	** < 0.001** ** < 0.001** 0.055 **0.031**
Age	1.049 (1.014–1.085)	**0.005**	1.020 (0.993–1.047)	0.148
Disease risk	2.281 (1.369–3.800)	**0.002**	2.168 (1.467–3.204)	** < 0.001**
Conditioning regimen	0.923 (0.322–2.641)	0.881	0.850 (0.364–1.988)	0.708
HLA-compatibility	1.950 (0.986–3.856)	0.055	1.678 (0.994–2.835)	0.053
Gender match	2.552 (1.545–4.214)	** < 0.001**	1.374 (0.921–2.051)	0.120

*p*-values of less than 0.05 indicated statistical significance (in bold)

*BU* busulfan, *I.v.* intravenous, *TDM* therapeutic drug monitoring, *HLA* human leukocyte antigen

## Discussion

This study demonstrates that the route of busulfan administration significantly effects TRM and OS in pediatric patients. We showed that i.v. administration of busulfan with TDM results in a significantly lower TRM rate for patients with malignant and non-malignant diseases compared to i.v. administration without TDM, who, in turn, had a better outcome than patients with oral administration of busulfan. Moreover, we demonstrated that i.v. administration of busulfan with TDM results in a significantly improved 5y-OS for patients with malignant and non-malignant diseases. There were no differences in disease entities regarding the route of busulfan administration. Earlier studies have already shown that i.v. administration leads to better survival outcomes than oral administration (Kashyap et al. 2002). One reason for this is the improved and more predictable bioavailability due to a more controlled administration. The gastrointestinal tract with less predictable absorption, is circumvented by i.v. administration (Kashyap et al. 2002), reducing PK variability. Furthermore, frequent complications of oral administration impacting PK such as vomiting can be avoided. Vomiting was a recurring problem upon oral administration of busulfan, making it notoriously difficult to retrace the ingested quantity. Many pediatric patients required repeated doses after vomiting, leading to the preference for i.v. administration. Our study shows that patients with i.v. administration had a lower incidence of TRM and an improved OS. Nonetheless, variability remains persistent even with i.v. administration. Particularly in young children, bioavailability varies due to a higher busulfan clearance (Oechtering et al. 2005). Additionally, young children are more likely to develop hepatic SOS, emphasizing the importance of controlled administration, and justifying the introduction of TDM for i.v. administration.

Patients with i.v. busulfan administration and additional TDM showed the best outcome concerning TRM and OS in our study. This could be explained by improved cumulative target AUCs: patients with low cumulative AUCs were identified in real-time and received individualized dose adjustments to correct cumulative AUCs, increasing efficacy and lowering the rate of relapse, mixed chimerism and graft failure. Ansari et al. (2014) showed that i.v. busulfan administration and particularly the first-dose PK is associated with better OS and event-free survival, and lower non-relapse mortality.

It is important to mention that we used different target AUCs based on the underlying disease. In patients with malignant diseases, we treated patients with higher cumulative target AUCs (85.0 mg/L x h) to ensure eradication of all cancer cells. This target AUC was established as a basis for the subsequent HSCT to achieve good outcomes and prevent relapses. In contrast, patients with non-malignant diseases were treated with lower cumulative target AUCs (70.0 mg/L x h). In these patients, busulfan was primarily used to eliminate the recipient’s hematopoiesis. In targeting lower cumulative AUCs, toxicity could be reduced (Chandra et al. 2021). Güngör et al. (2014) recommended a target AUC of 45–65 mg/L × h for patients with chronic granulomatous disease to avoid organ toxicity, which resembles the lower incidence of TRM demonstrated in our study. Additionally, monitoring is also relevant for reduced toxicity. Monitoring the plasma levels and thus calculating the AUC helps prevent overdosing and therefore adverse effects such as hepatic SOS. Esteves et al. (2020) claimed that patients with AUC levels greater than 5000 µmol*min/day (cumulative AUC of 82.1 mg/L x h) have a higher risk of developing hepatic SOS. Furthermore, Bognàr et al. (2022) showed that a cumulative AUC > 78 mg/L x h is associated with a higher risk of hepatic SOS in patients with only busulfan as one alkylator. The lower AUC cut-off of 78 mg/L x h compared to our cut-off AUC value of 85.0 mg/L x h for patients with malignant diseases may be explained by the underlying disease of the patients. Some diseases (e.g. osteopetrosis, hemophagocytic lymphohistiocytosis) have a higher risk of developing hepatic SOS. Moreover, additional factors can affect the risk of developing hepatic SOS (Felber et al. 2020; Kloehn et al. 2022).

Veal et al. (2012) demonstrated that pediatric patients with i.v. administration of busulfan showed a lower PK variability and are more likely to achieve target AUC values compared to children receiving oral busulfan administration. However, PK variability could not be eliminated entirely by i.v. administration. One reason for this could be the different other therapeutic agents used in conditioning regimens based on the different diagnoses, which may affect the PK of busulfan (Schreib et al. 2023).

Although we demonstrated that i.v. administration combined with TDM leads to the lowest incidence of TRM and the best OS, it remains unclear which target cumulative AUC best balances efficacy and toxicity. Nevertheless, Tesfaye et al. (2014) showed that in 68% of their patients, dose adjustments were necessary to achieve the target AUC, which emphasizes the importance of TDM in busulfan-based conditioning regimens. For even better outcomes, it is essential to define narrow AUC ranges for pediatric patients with both malignant as well as non-malignant diseases.

While our study shows promising results, it must be noted that this study is retrospective and a single-center study. Moreover, the estimation of TRM and OS for patients with malignant and non-malignant diseases could be improved if more patients were included in each group.

In summary, our study emphasizes the importance of i.v. administration of busulfan with TDM for pediatric patients to achieve lower incidence of TRM and improved OS. Patients with i.v. administration and TDM had a significantly lower incidence of TRM, and a significantly improved OS compared to patients with only i.v. busulfan administration, who, in turn, had a better outcome than patients with oral busulfan administration. Our study reinforces the relevance of TDM in patients with malignant diseases, and we recommend a cut-off cumulative AUC of < 85.0 mg/L x h to prevent hepatic SOS in these children. To validate our results, further studies are necessary.

## Data Availability

No datasets were generated or analysed during the current study.

## References

[CR1] Ansari M, Théoret Y, Rezgui MA, Peters C, Mezziani S, Desjean C, Vachon MF, Champagne MA, Duval M, Krajinovic M, Bittencourt H; Pediatric Disease Working Parties of the European Blood and Marrow Transplant Group (2014) Association between busulfan exposure and outcome in children receiving intravenous busulfan before hematopoietic stem cell transplantation. Ther Drug Monit 36(1):93–99 24061446 10.1097/FTD.0b013e3182a04fc7

[CR2] Arndt C, Beck JF, Gruhn B (2014) A pediatric prognostic score for patients undergoing allogeneic hematopoietic stem cell transplantation. Eur J Haematol 93(6):509–515 24889859 10.1111/ejh.12390

[CR3] Bartelink IH, Bredius RGM, Ververs TT, Raphael MF, Van Kesteren C, Bierings M, Rademaker CMA, den Hartigh J, Uiterwaal CSPM, Zwaveling J, Boelens JJ (2008) Once-daily intravenous busulfan with therapeutic drug monitoring compared to conventional oral busulfan improves survival and engraftment in children undergoing allogeneic stem cell transplantation. Biol Blood Marrow Transp 14(1):88–98 10.1016/j.bbmt.2007.09.015 18158965

[CR4] Bartelink IH, Lalmohamed A, van Reij EML, Dvorak CC, Savic RM, Zwaveling J, Bredius RGM, Egberts ACG, Bierings M, Kletzel M, Shaw PJ, Nath CE, Hempel G, Ansari M, Krajinovic M, Théorêt Y, Duval M, Keizer RJ, Bittencourt H, Hassan M, Güngör T, Wynn RF, Veys P, Cuvelier GDE, Marktel S, Chiesa R, Cowan MJ, Slatter MA, Stricherz MK, Jennissen C, Long-Boyle JR, Boelens JJ (2016) Association of busulfan exposure with survival and toxicity after haemopoietic cell transplantation in children and young adults: a multicentre, retrospective cohort analysis. Lancet Haematol 3(11):e526–e536 27746112 10.1016/S2352-3026(16)30114-4 PMC5159247

[CR5] Benadiba J, Ansari M, Krajinovic M, Vachon MF, Duval M, Teira P, Cellot S, Bittencourt H (2018) Pharmacokinetics-adapted Busulfan-based myeloablative conditioning before unrelated umbilical cord blood transplantation for myeloid malignancies in children. PLoS ONE 13(4):e0193862 29608607 10.1371/journal.pone.0193862 PMC5880335

[CR6] Bognàr T, Bartelink IH, Egberts TCG, Rademaker CMA, Versluys AB, Slatter MA, Kletzel M, Nath CE, Cuvelier GDE, Savic RM, Dvorak C, Long-Boyle JR, Cowan MJ, Bittencourt H, Bredius RGM, Güngör T, Shaw PJ, Ansari M, Hassan M, Krajinovic M, Hempel G, Marktel S, Chiesa R, Théoret Y, Lund T, Orchard PJ, Wynn RF, Boelens JJ, Lalmohamed A (2022) Association Between the Magnitude of Intravenous Busulfan Exposure and Development of Hepatic Veno-Occlusive Disease in Children and Young Adults Undergoing Myeloablative Allogeneic Hematopoietic Cell Transplantation. Transp Cell Ther 28(4):196–202 10.1016/j.jtct.2022.01.013 35065280

[CR7] Bolinger AM, Zangwill AB, Slattery JT, Glidden D, DeSantes K, Heyn L, Risler LJ, Bostrom B, Cowan MJ (2000) An evaluation of engraftment, toxicity and busulfan concentration in children receiving bone marrow transplantation for leukemia or genetic disease. Bone Marrow Transp 25(9):925–930 10.1038/sj.bmt.1702371 10800058

[CR8] Chandra S, Chandrakasan S, Dávila Saldaña BJ, Bleesing JJ, Jordan MB, Kumar AR, Grimley MS, Krupski C, Davies SM, Khandelwal P, Marsh RA (2021) Experience with a Reduced Toxicity Allogeneic Transplant Regimen for Non-CGD Primary Immune Deficiencies Requiring Myeloablation. J Clin Immunol 41(1):89–98 33067658 10.1007/s10875-020-00888-2 PMC8406429

[CR9] Chattergoon DS, Saunders EF, Klein J, Calderwood S, Doyle J, Freedman MH, Koren G (1997) An improved limited sampling method for individualised busulphan dosing in bone marrow transplantation in children. Bone Marrow Transp 20(5):347–354 10.1038/sj.bmt.1700891 9339748

[CR10] Corbacioglu S, Carreras E, Ansari M, Balduzzi A, Cesaro S, Dalle JH, Dignan F, Gibson B, Guengoer T, Gruhn B, Lankester A, Locatelli F, Pagliuca A, Peters C, Richardson PG, Schulz AS, Sedlacek P, Stein J, Sykora KW, Toporski J, Trigoso E, Vetteranta K, Wachowiak J, Wallhult E, Wynn R, Yaniv I, Yesilipek A, Mohty M, Bader P (2018) Diagnosis and severity criteria for sinusoidal obstruction syndrome/veno-occlusive disease in pediatric patients: a new classification from the European society for blood and marrow transplantation. Bone Marrow Transp 53(2):138–145 10.1038/bmt.2017.161 PMC5803572 28759025

[CR11] Esteves I, Santos FPS, Ribeiro AAF, Seber A, Sugawara EK, Sobrinho JJDN, Barros JC, Oliveira JSR, Fernandes JF, Hamerschlak N, Andersson BS, de Lima M, Kerbauy FR (2020) Targeted-dose of busulfan: Higher risk of sinusoidal obstructive syndrome observed with systemic exposure dose above 5000 µMol min. A historically controlled clinical trial. Hematol Oncol 38(5):773–781 32779746 10.1002/hon.2789

[CR12] Felber M, Steward CG, Kentouche K, Fasth A, Wynn RF, Zeilhofer U, Haunerdinger V, Volkmer B, Prader S, Gruhn B, Ehl S, Lehmberg K, Müller D, Gennery AR, Albert MH, Hauck F, Rao K, Veys P, Hassan M, Lankester AC, Schmid JP, Hauri-Hohl MM, Güngör T (2020) Targeted busulfan-based reduced-intensity conditioning and HLA-matched HSCT cure hemophagocytic lymphohistiocytosis. Blood Adv 4(9):1998–2010 32384542 10.1182/bloodadvances.2020001748 PMC7218427

[CR13] Feng X, Wu Y, Zhang J, Li J, Zhu G, Fan D, Yang C, Zhao L (2020) Busulfan systemic exposure and its relationship with efficacy and safety in hematopoietic stem cell transplantation in children: a meta-analysis. BMC Pediatr 20(1):176 32312247 10.1186/s12887-020-02028-6 PMC7168843

[CR14] Grochow LB, Krivit W, Whitley CB, Blazar B (1990) Busulfan disposition in children. Blood 75(8):1723–1727 2328321

[CR15] Güngör T, Teira P, Slatter M, Stussi G, Stepensky P, Moshous D, Vermont C, Ahmad I, Shaw PJ, Telles da Cunha JM, Schlegel PG, Hough R, Fasth A, Kentouche K, Gruhn B, Fernandes JF, Lachance S, Bredius R, Resnick IB, Belohradsky BH, Gennery A, Fischer A, Gaspar HB, Schanz U, Seger R, Rentsch K, Veys P, Haddad E, Albert MH, Hassan M; Inborn Errors Working Party of the European Society for Blood and Marrow Transplantation (2014) Reduced-intensity conditioning and HLA-matched haemopoietic stem-cell transplantation in patients with chronic granulomatous disease: a prospective multicentre study. Lancet 383(9915):436–448 24161820 10.1016/S0140-6736(13)62069-3

[CR16] Kashyap A, Wingard J, Cagnoni P, Roy J, Tarantolo S, Hu W, Blume K, Niland J, Palmer JM, Vaughan W, Fernandez H, Champlin R, Forman S, Andersson BS (2002) Intravenous versus oral busulfan as part of a busulfan/cyclophosphamide preparative regimen for allogeneic hematopoietic stem cell transplantation: decreased incidence of hepatic venoocclusive disease (HVOD), HVOD- related mortality, and overall 100-day mortality. Biol Blood Marrow Transp 8(9):493–500 10.1053/bbmt.2002.v8.pm12374454 12374454

[CR17] Kloehn J, Brodt G, Ernst J, Gruhn B (2022) Analysis of risk factors for hepatic sinusoidal obstruction syndrome following allogeneic hematopoietic stem cell transplantation in pediatric patients. J Cancer Res Clin Oncol 148(6):1447–1455 34255148 10.1007/s00432-021-03732-1 PMC9114040

[CR18] Malär R, Sjöö F, Rentsch K, Hassan M, Güngör T (2011) Therapeutic drug monitoring is essential for intravenous busulfan therapy in pediatric hematopoietic stem cell recipients. Pediatr Transp 15(6):580–588 10.1111/j.1399-3046.2011.01529.x 21736681

[CR19] McCune JS, Holmberg LA (2009) Busulfan in hematopoietic stem cell transplant setting. Expert Opin Drug Metab Toxicol 5(8):957–969 19611402 10.1517/17425250903107764

[CR20] McCune JS, Gibbs JP, Slattery JT (2000) Plasma concentration monitoring of Busulfan. Does it improve clinical outcome? Clini Pharmacokinet 39(2):155–165 10.2165/00003088-200039020-00005 10976660

[CR21] Nguyen L, Fuller D, Lennon S, Leger F, Puozzo C (2004) I.V. busulfan in pediatrics: a novel dosing to improve safety/efficacy for hematopoietic progenitor cell transplantation recipients. Bone Marrow Transplant 33(10):979–987 15064687 10.1038/sj.bmt.1704446

[CR22] Oechtering D, Schiltmeyer B, Hempel G, Schwab M, Würthwein G, Mürdter T, Klingebiel T, Vormoor J, Gruhn B, Fleischack G, Boos J (2005) Toxicity and pharmacokinetics of i.v. busulfan in children before stem cell transplantation. Anticancer Drugs 16(3):337–344 15711187 10.1097/00001813-200503000-00014

[CR23] Santos GW, Tutschka PJ, Brookmeyer R, Saral R, Beschorner WE, Bias WB, Braine HG, Burns WH, Elfenbein GJ, Kaizer H et al (1983) Marrow transplantation for acute nonlymphocytic leukemia after treatment with busulfan and cyclophosphamide. N Engl J Med 309(22):1347–1353 6355849 10.1056/NEJM198312013092202

[CR24] Schiltmeyer B, Klingebiel T, Schwab M, Mürdter TE, Ritter CA, Jenke A, Ehninger G, Gruhn B, Würthwein G, Boos J, Hempel G (2003) Population pharmacokinetics of oral busulfan in children. Cancer Chemother Pharmacol 52(3):209–216 12811512 10.1007/s00280-003-0631-y

[CR25] Schreib KM, Bräm DS, Zeilhofer UB, Müller D, Güngör T, Krämer SD, Hauri-Hohl MM (2023) Population Pharmacokinetic Modeling for Twice-Daily Intravenous Busulfan in a Large Cohort of Pediatric Patients Undergoing Hematopoietic Stem Cell Transplantation-A 10-Year Single-Center Experience. Pharmaceutics 16(1):0 10.3390/pharmaceutics16010013 PMC11154452 38276491

[CR26] Scott LJ, Hoy SM, Lyseng-Williamson KA (2012) Intravenous busulfan: a guide to its use as conditioning treatment before transplantation of haematopoietic progenitor cells. Clin Drug Investig 32(9):641–648 22877323 10.1007/BF03261918

[CR27] Shao DF, Li JH, Hu T, Zhang ZX, Zhang L, Li JJ, Cao J, Feng SQ, Tang RH, Zhong DX, Song ZL, Yue M, Hu MZ, Xuan LT, Zhai MN, Zhang HF, Wang XY, Shi XD, Liu R (2022) Clinical outcomes of individualized busulfan-dosing in hematopoietic stem cell transplantation in Chinese children undergoing with therapeutic drug monitoring. Bone Marrow Transp 57(3):473–478 10.1038/s41409-021-01545-x 35039622

[CR28] Shimoni A, Bielorai B, Toren A, Hardan I, Avigdor A, Yeshurun M, Ben-Bassat I, Nagler A (2003) Intravenous busulfan-based conditioning prior to allogeneic hematopoietic stem cell transplantation: myeloablation with reduced toxicity. Exp Hematol 31(5):428–434 12763142 10.1016/s0301-472x(03)00047-x

[CR29] Socié G, Clift RA, Blaise D, Devergie A, Ringden O, Martin PJ, Remberger M, Deeg HJ, Ruutu T, Michallet M, Sullivan KM, Chevret S (2001) Busulfan plus cyclophosphamide compared with total-body irradiation plus cyclophosphamide before marrow transplantation for myeloid leukemia: long- term follow-up of 4 randomized studies. Blood 98(13):3569–3574 11739158 10.1182/blood.v98.13.3569

[CR30] Tesfaye H, Branova R, Klapkova E, Prusa R, Janeckova D, Riha P, Sedlacek P, Keslova P, Malis J (2014) The importance of therapeutic drug monitoring (TDM) for parenteral busulfan dosing in conditioning regimen for hematopoietic stem cell transplantation (HSCT) in children. Ann Transplant 19:214–224 24811685 10.12659/AOT.889933

[CR31] Veal GJ, Nguyen L, Paci A, Riggi M, Amiel M, Valteau-Couanet D, Brock P, Ladenstein R, Vassal G (2012) Busulfan pharmacokinetics following intravenous and oral dosing regimens in children receiving high-dose myeloablative chemotherapy for high-risk neuroblastoma as part of the HR-NBL-1/SIOPEN trial. Eur J Cancer 48(16):3063–3072 22742881 10.1016/j.ejca.2012.05.020

[CR32] Zao JH, Schechter T, Liu WJ, Gerges S, Gassas A, Maarten Egeler R, Grunebaum E, Lee Dupuis L (2015) Performance of Busulfan Dosing Guidelines for Pediatric Hematopoietic Stem Cell Transplant Conditioning. Biol Blood Marrow Transp 21(8):1471–1478 10.1016/j.bbmt.2015.05.006 25977229

